# The Promise and Perils of Artificial Intelligence in Advancing Participatory Science and Health Equity in Public Health

**DOI:** 10.2196/65699

**Published:** 2025-02-14

**Authors:** Abby C King, Zakaria N Doueiri, Ankita Kaulberg, Lisa Goldman Rosas

**Affiliations:** 1 Departments of Epidemiology & Population Health and of Medicine (Stanford Prevention Research Center), Stanford University School of Medicine, 1701 Page Mill Road, Palo Alto, CA, 94304-1210, United States, 1 650-497-2806; 2 Department of Epidemiology & Population Health, Stanford University School of Medicine, Palo Alto, CA, United States; 3 Departments of Epidemiology & Population Health and of Medicine (Division of Primary Care and Population Health), Stanford University School of Medicine, Stanford University, Palo Alto, CA, United States

**Keywords:** digital health, artificial intelligence, community-based participatory research, citizen science, health equity, societal trends, public health, viewpoint, policy makers, public participation, information technology, micro-level data, macro-level data, LLM, natural language processing, machine learning, language model, Our Voice

## Abstract

Current societal trends reflect an increased mistrust in science and a lowered civic engagement that threaten to impair research that is foundational for ensuring public health and advancing health equity. One effective countermeasure to these trends lies in community-facing citizen science applications to increase public participation in scientific research, making this field an important target for artificial intelligence (AI) exploration. We highlight potentially promising citizen science AI applications that extend beyond individual use to the community level, including conversational large language models, text-to-image generative AI tools, descriptive analytics for analyzing integrated macro- and micro-level data, and predictive analytics. The novel adaptations of AI technologies for community-engaged participatory research also bring an array of potential risks. We highlight possible negative externalities and mitigations for some of the potential ethical and societal challenges in this field.

## Introduction

“While the future might indeed be bright for AI, it wouldn’t be so by accident. We will have to earn it, together.”

Dr. Fei-Fei Li

Founding Director, Stanford Institute for Human-Centered AI

From *The Worlds I See: Curiosity, Exploration, and Discovery at the Dawn of AI* [1]

The last several decades have witnessed a growing mistrust in science among both policy makers and the public at large on an unprecedented scale [2]. The reasons for this trend appear to be many and varied, including a lack of understanding about how science actually operates, partly at least due to an absence of participatory educational opportunities and direct engagement in meaningful science activities [3]. This, in turn, has been linked with poor self-ratings of scientific literacy among school-aged adolescents and adults alike [3]. A general confusion about what the public should reasonably expect from research in terms of scientific veracity or “truth” has been an additional contributor to the public’s skepticism about science. These problems have been amplified to a significant extent by social media, which has often worked, either intentionally or unintentionally, to confuse or obfuscate the truth [4]. Unfortunately, such efforts arguably have been abetted by scientific institutions themselves as well as scientific journal outlets which, in their haste to grab the day’s headlines, have at times stripped scientific results of their complexity, nuance, and context; or, shockingly, filled untold numbers of journals with “fake science” [5]. Well-documented historical injustices also have contributed to a lack of trust in academic and scientific institutions, particularly among communities of color. For example, the US Public Health Service Untreated Syphilis Study at Tuskegee has generated mistrust in health care institutions and research for generations [6]. More recently, the University of Arizona was found guilty of using DNA samples from the Havasupai Tribe for research without their consent [7]. Recent controversies and public health crises (eg, the COVID-19 pandemic) also have given rise to increased questioning of public health authorities and have sown doubts regarding scientific validity and reliability, which have exacerbated feelings of mistrust.

This alarming trend of mistrust in science threatens public health efforts in multiple ways, which include the following recent examples: (1) misinformation concerning vaccines, including outright dismissal of the extensive multigenerational evidence base supporting the development and use of vaccines to prevent a myriad of dangerous and at times fatal diseases that have threatened the public’s health; (2) skepticism surrounding the wide-ranging, multidisciplinary evidence base establishing the considerable effects of climate change that threaten the health of the planet and all of its living inhabitants; and (3) the growing distrust of health care systems as well as pharmaceutical companies (ie, “big Pharma”), which has become especially acute among marginalized groups, including minoritized and under-resourced communities [8].

The mistrust of science coincides with other pernicious societal trends reflecting disconnection and skepticism in other facets of daily life. These include growing concerns in the health and mental health fields around the current levels of civic disconnectedness and community disengagement among significant sections of the population. Recent data indicate, for example, that civic participation, social engagement, and community connection among segments of today’s youth and young adults are declining, with this trend having been worsened by the COVID-19 pandemic [9].

The confluence of these trends with the emergence of an often-bewildering pace of advances in the worldwide IT sector has served to exacerbate feelings of confusion and mistrust among broad segments of the population. This has included, in particular, the unexpectedly disruptive force of the current artificial intelligence (AI) revolution across all sectors, including health care, education, and research. Threats regarding the spread of misinformation, increasing systematic biases against already marginalized populations, data privacy issues, and potential job displacement are among the leading concerns of the public, in addition to other impacts that the widespread acceptance and use of AI might have on various segments of society, from individuals through governments and policy makers [10].

The above issues have led to the following questions: (1) how can science in general be made more accessible to the public in ways that can reduce confusion and mistrust and increase the meaningfulness of scientific inquiry to directly benefit individuals and their communities, and (2) how can the potential of AI be harnessed to bolster the public’s engagement in public health science while mitigating the distrust and confusion about both research and AI itself?

## Increasing Public Participation in Scientific Research

Public participation in science research (PPSR) is one way researchers have been addressing the increasing mistrust among decision makers and the public toward science. A growing number of studies have included PPSR methods, such as citizen science, that have utilized educational outreach efforts to increase public knowledge and awareness of scientific principles and best practices [11]. Important, systematic efforts have been employed to actively involve a greater number and variety of community members in the scientific process [12]. Such participatory research paradigms can have substantial positive impacts for increasing transparency, knowledge, and trust in scientific inquiry, particularly when they involve generating actionable data of direct relevance to those involved [13].

Within the broader umbrella of PPSR frameworks, citizen science—generally defined as involving lay persons in the research process to advance science—is an increasingly popular approach to engage community members in research activities [14]. A strength behind citizen science is its underlying principle that scientific inquiry can often benefit from including diverse members of the community from varying educational, occupational, social, and cultural backgrounds. Various forms of citizen science have occurred over several centuries and have involved diverse fields, including the life, environmental, health, social, and behavioral sciences [15]. Citizen science can directly benefit research by providing extra pairs of “helping hands” in the data collection and problem-solving aspects of science [14]. Beyond democratizing data acquisition, citizen scientists also can contribute to the fuller research process, including problem definition, data interpretation and prioritization, data-driven solution generation, and results dissemination [16-18]. This more fully participatory “by the people” form of citizen science can advance scientific inquiry as well as promote meaningful knowledge and solutions for the local communities that are involved [13,16,17].

An example of this type of citizen science in the public health arena is the technology-enabled “Our Voice” Global Citizen Science Research Initiative [19], where residents from diverse and often under-resourced communities learn how to partner with researchers and community organizations in collecting and interpreting aggregated and anonymized contextual data (eg, photos, text, and audio). These data identify features of residents’ local environments that help or hinder their health [16,17]. They then learn how to effectively communicate their data to decision makers and work with them to activate health-benefiting changes in their local environments [16,17]. Over nearly a decade, the multi-generational “Our Voice” Initiative has produced demonstrable community-driven physical and social environmental impacts across 100+ projects involving over 25 countries spanning 6 continents [16,17].

## Leveraging the Potential of AI to Promote More Powerful Citizen Science for Advancing Public Health

**Figure 1.**
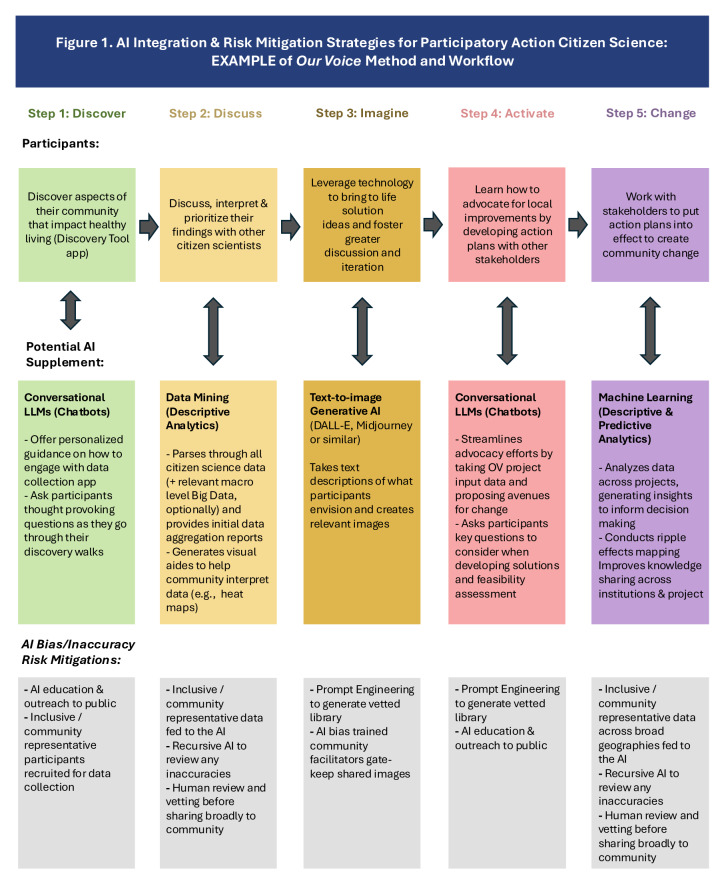
Artificial intelligence (AI) integration and risk mitigation strategies for participatory action citizen science: example of “Our Voice” Method and workflow. LLM: large language model; OV: Our Voice.

While the current focus of health-oriented AI applications has been primarily at the individual patient level, the thoughtful and strategic application of AI at the community level offers potentially powerful tools to augment participatory science activities across the fuller research process. At the same time, it is important that both researchers and participants recognize the potential risks of AI and are committed to implementing multilayered mitigation tactics aimed at addressing them (see Table 1, Figure 1, and the subsequent section focused on risk mitigation).

**Table 1.** Examples of potential artificial intelligence (AI) uses within community-engaged public health citizen science research, along with potential risks and risk mitigation strategies.

Potential use of AI within community-engaged research	Potential risks	Risk mitigation strategies
Conversational LLMs^a^ (ChatGPT or similar) for onboarding participants, offering personalized guidance on engaging with technology, and asking thought-provoking questions	Biased or culturally insensitive conversations emerge AI “hallucinations” where factually inaccurate commentary is made	Dedicated time spent in prompt engineering to determine language best used to minimize these biases in this context, and then shared as best practices to the public Public education on AI, the training data that were used, and discussion of shortcomings
Text-to-image Generative AI tools (DALL-E, Midjourney, or similar) for helping the community visualize their data (eg, turning text descriptions into relevant images)	Biased or culturally insensitive pictures generated and shared Impossible or impractical to execute ideas presented	Human (expert) review and gatekeeping before images shared broadly to the public Prompt engineering and similar efforts aimed at community data collection are shared with participants in advance to enhance the potential relevance and feasibility of solutions
Data Mining (Descriptive Analytics) for analyzing macro- and microlevel data, generating integrated data reports, visualizations, etc.	Inaccuracies or AI hallucinations injected into the dataset Loss of control, research rigor, and cognitive competence by scientists who defer too much to AI	Intentional efforts made to feed AI inclusive and community representative data Recursive AI use: feed output back to AI to check for inaccuracies Require scientists to review all data generated for accuracy and meet field standards for scientific literacy or competence
Data Mining (Predictive Analytics) for reviewing large datasets, providing data aggregation reports, and generating visual aids to help the community interpret data	Data presented may result in wrong conclusions, resulting in further inequities Loss of control, research rigor, and cognitive competence by scientists who defer too much to AI	Human (expert) review and governance of all generated findings Recursive AI use: feed output back to AI to check for inaccuracies Require scientists to review all data and methods for accuracy and meet field standards for scientific literacy or competence Empirical testing of AI to determine when it helps or hurts scientific skills and competencies

a LLM: large language model

Using the “Our Voice” multistep citizen science method as an example, potentially valuable AI applications can occur at each step (see Figure 1). The steps include resident-engaged data collection using a multilingual mobile app (“Discover” step); facilitated analysis, discussion, and prioritization of relevant local issues (“Discussion” step); leveraging of visualization technologies to bring potential solutions into clearer focus (“Imagine” step); communicating relevant community issues and brainstorming feasible solutions with local decision makers (“Activate” step); and continued partnering with stakeholders in bringing action plans to fruition (“Change” step). In addition to these integral steps, the above “Our Voice” method has been supplemented at times with an increasingly proactive and thoughtful approach to engage the community prior to embarking on citizen science activities, as well as the thoughtful dissemination of findings and insights to researchers, decision makers, and the public (a “Share” step). The careful integration of relevant AI applications into these different citizen science processes may assist in supplementing and enriching the different step outputs, as well as helping to address at least some of the challenges with public trust, given the active role that community members play in this type of participatory science.

Some examples of potentially relevant AI tools that can be explored are given below for each step.

### Discover (Data Collection) Step

A challenge to address in the discover (data collection) step is that the inclusion of AI in this step should not supersede the real, on-the-ground data collected by local citizen scientists. Any additional data presented by the AI at this step is intended to be an augmentation that goes through human review before it is included in project data capture.

Conversational large language models (LLMs) can be employed in a strategic and transparent way to personally guide the use of citizen science data collection apps such as the “Our Voice Discovery Tool” app, along with “real-time” dynamic prompts to more fully engage residents as they go through their data collection walks. In addition, utilizing computer vision, hearing, and other “real-world” perceptual sensing tools (eg, smell) may help to produce more robust and comprehensive data capture and classifications to augment the contextually focused citizen science data [20]. The goal of using such sensing tools along with LLMs would be to enrich citizen scientists’ data collection activities to produce more nuanced and detailed data to analyze and combine with other relevant data sources (eg, geographic information systems and social network models).

### Discuss Step

A challenge to address in the discuss step is that AI should not absolve researchers and participants from the task of ensuring analytical accuracy and rigor, nor replace efforts in interpreting the data and gleaning insights. As AI is known at times to experience “hallucinations” and commit errors when it comes to data analysis and interpretation, whatever is generated by it should be seen as a first-pass draft of something that helps researchers and community members enrich their own interpretations and iterations.

ChatGPT and similar conversational LLMs can be engaged during community meetings to support residents’ group discussions during data interpretation and solution-building activities. For example, LLMs may be able to support thoughtful community-level discussion and advocacy, not as an omniscient “black box” but as an attentive community-level support system and Socratic thinking aide. Rather than attempting to “replace” community members’ perspectives and lived experiences, LLMs may be employed to play the role of “devil’s advocate” in a more neutral way, to help residents better understand the concerns of marginalized groups as well as policy makers. LLMs also may allow participants to better assess the feasibility of their data-driven ideas for positive change by supplementing knowledge or experience gaps that the average citizen scientist may have (eg, “Considering the financial constraints of X and timeframe to solve this issue by Y, which of these 5 solutions are the most feasible to address …..?”). In presenting participants with more detailed background information regarding the implementation of different data-driven solutions or strategies, residents can be further informed about the potential complexities that come with improving their communities. AI also could be leveraged to generate and describe multilevel insights by combining diverse sources of data, from citizen scientist–generated microscale data that capture local contexts and individual-level biosensing outputs (eg, heart rate and physiological stress responses) through macroscale population-based social determinants and health data. This, in turn, could result in the development of interactive maps from such mixed-methods data sources that arguably could foster faster insights and public health responses from policy makers. For example, the initial efforts to visually combine citizen scientist–generated microscale data describing residents’ neighborhood-lived experiences with macroscale epidemiological data about their larger environmental contexts have yielded richer results than would have been obtained from either data source alone [21]. Leveraging AI in this manner to assist with combining and visualizing different forms of data could actually free up researchers and citizen scientists to engage in higher-order thinking and analysis that is required for creatively addressing some of society’s most pressing issues. For instance, in California, low participation in community-supported public health initiatives that provide opportunities for affordable healthy food access is often driven by diverse factors that can vary by locale. The multifactorial issues driving low program uptake often are challenging for researchers, governmental agencies, and community-based organizations to understand sufficiently to be able to intervene effectively. It is possible that AI tools could help by aiding the visualization of how microlevel “lived experience” data vary with respect to macrolevel contextual data. Such multilevel visualizations would allow researchers and community partners to spend more of their time understanding and solving the problem, as opposed to gathering and synthesizing relevant data to describe the problem.

### Imagine Step

A challenge to address in the imagine step is that AI should not have free reign in determining what the best solutions for the community are. Using culturally tailored inputs, AI’s creativity can be used as a supplemental aide to the collective community’s brainstorming while not superseding it.

Several AI tools may be useful in helping residents, decision makers, and researchers better imagine what scientifically generated health-enhancing changes can actually look like in their own communities and environments (eg, through generative AI text-to-image tools such as DALL-E and Midjourney). For example, Stanford’s Center on Human-Centered Artificial Intelligence awarded a seed grant to “Our Voice” that will allow citizen scientists to take images collected during local “discovery walks” and transform them into reimagined futures through such generative AI tools (eg, an image of an unsafe intersection to get to school could be “morphed” into images of different solutions such as a crosswalk, pedestrian bridge, etc). The early testing of this form of AI with school children living in the El Pozón neighborhood of Cartagena, Colombia—where the majority of inhabitants live below the poverty line and lack access to essential services—is showing how the thoughtful, culturally tailored use of text-to-image AI tools can generate meaningful and actionable solutions to local environmental problems (eg, stagnant water and uncollected garbage). These rapid visual renderings could in turn lead to enhanced and richer discussions of trade-offs and foster new ideas among community members and researchers.

### Activate and Change Steps

A challenge to address in the activate and change steps is that, as noted above, an optimal goal of AI use is to supplement and enrich such conversations, as opposed to driving them. Sharing the above types of visualization tools and information with local decision makers may help to jump-start relevant solution-building efforts. Generative AI could be used to aid in the creation of presentation materials, help community members role-play conversations with local decision makers in preparation for scheduled meetings with them, and identify issues and suggest modifications to the action plan created during the community meetings.

### Share Step

A challenge to address in the share step is that it is recommended that researchers avoid risking diminished scientific rigor and accuracy through outsourcing major data interpretation to AI. As noted earlier, people are in the best position to fully understand and evaluate what the data mean, including, in this case, the citizen scientists who have collected those data.

Machine learning tools also can be used to aid researchers in the aggregation of data across projects and expedite their ability to glean useful insights across different citizen science projects and research groups.

In addition to the above types of citizen science-oriented strategies, through utilizing AI-generated virtual reality simulations, citizen scientists along with other community members can experience physical, cultural, and social situations that they might not otherwise encounter in their daily lives [22]. Such AI-generated virtual reality simulations also can provide community members and decision makers with hands-on opportunities to interact with virtual characters representing differing cultural backgrounds that may lead to increased cultural sensitivity [22]. These AI-driven virtual or simulated environments can additionally allow community members to try out citizen science data gathering and similar tools before real-world use [20].

## Additional Recommendations on Mitigating Potential Risks of AI

Amidst the potentially promising uses of AI for the public health and citizen science fields noted above, including its potential to expand the breadth and utility of the data being collected and interpreted, a number of ethical challenges and concerns have been raised, some of which have been touched on earlier and in other articles [23]. These include the different types of risks, as described in this paragraph. AI could diminish, distort, or replace data being collected in the field with output that is less accurate (ie, AI “hallucinations”) and less representative of community member experiences and responses. AI output may be based on information and sources that are not culturally relevant or appropriate for a given community or context. Potential challenges emerge when AI is presented with contrasting ideas from citizen scientists, researchers, and other stakeholders and autonomously makes prioritization choices among them. The introduction of AI tools into the public health research process could actually serve to increase the “depersonalization” of the research process, leading to a greater rather than a lower distrust of science. Finally, major concerns have been raised that through potentially oversimplifying and directing aspects of the scientific process, LLMs and similar AI tools could significantly diminish scientists’ data processing skills and cognitive competencies.

The publicly available use of ChatGPT and similar generative AI tools as “short cuts” for increasing time efficiencies at the potential expense of thought and deliberation has served to heighten many of these concerns.

In response to such threats, some potential solutions have begun to emerge (see Table 1), as described below.

### Thoughtfulness and Transparency

A potential solution is being thoughtful and transparent in determining when IA (information augmentation to support human intelligence) versus AI (artificial intelligence, which is often used as a replacement for some human activities) is best suited. For example, in places where the radius for impact is large and missteps would be difficult to rectify or could cause grave harm, AI, which to date generally does not have community participation or checks for bias mitigation, ought to be used with great caution. In such instances, using technology instead to aggregate and synthesize data findings, and leaving the interpretation of those findings to people, could be wiser.

A mitigation example could be the data collection of citizen scientists’ lived experiences in the context of community determinants of health that could be synthesized to share summary statistics, generate heat maps, or word clouds; however, any interpretation of their data, along with solution-building and implementation, would not be outsourced to AI without human supervision and community-level interpretations and buy-in.

### Employing Explainable AI Concepts and Strategies

Another potential solution is the use of explainable AI (XAI) that involves processes and methods that allow human users to understand and better trust the results and output created by machine learning and similar algorithms. It can be used to describe an AI model, its expected uses and impacts, and potential biases (eg, based on sex or race).

A mitigation example is the use of a set of vetted chat prompts and outlined best practices that could be provided to users (“prompt engineering”), instead of assuming that the results of ChatGPT are correct and accurate. These best practices could include examples of wording to enter into ChatGPT to improve results and ideally catch AI “hallucinations.” Here is one such example: “For any and all responses, end each response with a bulleted list that includes (1) any assumptions you are making, (2) citations of any sources you have used to determine the response to my prompt, and (3) any biases or concerns you want to review if you had access to unlimited data or personnel.” In addition, the responses generated by the AI system can be fed back to itself with the prompt “Check this response for any factual inaccuracies or state anything you cannot back up with reputable sources.”

### Launching Active Educational and Outreach Initiatives

There are growing concerns related to the potential of AI, including generative AI, to promote biased results and intensify health inequities through the lack of diverse data that are representative of different societal groups. Therefore, it is important to launch active educational and outreach initiatives to better inform the public across all walks of life about AI, its uses, ethical implications, and the precautions about which people should be aware. Educational efforts aimed specifically at diverse communities and populations may help to increase transparency concerning both the promise and limitations of this emerging field. It also may help ensure that key community norms and values are taken into account [24]. Such outreach-based multidisciplinary and community-driven educational efforts tailored to the needs of different populations may additionally help to empower currently under-represented groups to actively participate in data collection and information sharing to help mitigate such biases [24]. Involving community members in the development of AI methodologies from the beginning is important to ensure transparency and reduce mistrust among participants [24]. While such proactive educational efforts have been focused primarily on health care, they could benefit public health research and activities as well.

A mitigation example is described here. Zainab Garba-Sani and colleagues have developed the A.C.C.E.S.S. AI model, which lays a framework for involving communities in the AI-rollout process as follows: A = “Affirm your aims,” C = “Consider your communities,” C = “Cultivate your conversations,” E = “Embrace your essentials,” S = “Specify your scope,” and S = “Scrutinize your spaces” [24]. Through training sessions and workshops with diverse groups, A.C.C.E.S.S. serves the dual purpose of not only educating people about AI and its potential applications but also gathering their input on how AI should be thoughtfully designed for their unique contexts. Another example of such an initiative is the nonprofit “AI4ALL” movement that aims to advance an increasingly human-centered, inclusive AI discipline [25]. Through proactive participatory approaches such as these, we can begin to demystify AI and ensure that diverse communities are brought to the table when implementing AI tools.

### Increasing Efforts to Improve Data Upon Which AI is Modeled and Trained

As AI is merely holding up a mirror to the society or data upon which it is trained, the biases inherent in AI responses are likely due to the missing perspectives and incomplete data upon which it was built. Leveraging collaborations across institutions, social or civic interest groups, and diverse communities to improve the training data should help AI become more aware of differing perspectives and hopefully reduce if not completely minimize the bias. Outreach-based multidisciplinary and community-driven educational efforts as described below that are tailored to the needs of different populations may additionally help to empower currently under-represented groups to actively participate in data collection and information sharing to help mitigate such biases [24].

A mitigation example is the need for clear guidelines and incentives from scientific organizations, universities, journals, and granting organizations (eg, specific grant announcements from the NIH in this area) concerning expanding the population representativeness of the datasets upon which AI is based; this can send a clear message regarding the importance of this issue to the field as well as society at large.

### Setting AI-Specific Ethical Standards and Having Expected AI-Influenced Outputs Verified by Experts

It is important to set AI-specific ethical standards across universities, other research organizations, scientific outlets, and funding sources that include the ethics review of study methods as well as have the expected AI-influenced outputs verified by experts trained in the relevant ethics fields. Publishers of scientific journals, books, and other scientific communication channels should be encouraged to provide specific guidelines related to the publication of research investigations that include AI. Demanding transparency of AI use in scientific research more generally is recommended, as a growing collection of policy makers, technology developers, and scientific organizations are currently doing. Setting up appropriate “guardrails” prior to AI use is similarly important to help ensure that flawed, offensive, or inaccurate information does not unexpectedly find its way into AI-driven conversations or other outputs.

A mitigation example is described here. As part of institutional review board policies and activities, some grant-making organizations have included, in addition to the review of a grant proposal’s scientific merit, a separate ethics review involving experts in ethics and similar fields to identify areas of potential ethical concern and methods for the mitigation of such concerns. An example is the Stanford Human-Centered Artificial Intelligence Center’s process of including a separate ethics review of AI grant proposals to help ensure that appropriate AI-relevant guardrails are in place prior to funding.

### Regulating Transparent Data Ownership and Use

It is important to regulate transparent data ownership and use to ensure that access to citizen science data is not limited by commercial interests [20]; this helps to ensure that AI tools and resources are available to everyone, which can democratize its use and diminish public suspicion and skepticism. For example, many users of ChatGPT and similar platforms may not understand that their data currently may be sent to the parent company OpenAI to subsequently power future AI learning, and then be ultimately used by unnamed sources for a variety of undisclosed purposes.

A mitigation example is that, instead of relying on public GPTs whose data ownership and use are ambiguous, the Stanford School of Medicine and other academic institutions have developed their own custom GPT that ensure data security, HIPAA compliance, and similar outcomes. Additional AI resources that are being developed at Stanford include a user-friendly “AI Playground” platform, built on open-source technologies, that allows the Stanford community to try out various AI models and tools as well as compare the utility of different tools in a head-to-head fashion.

### Human Governance and Oversight Over AI Output

It is imperative when undertaking community-engaged forms of research that scientists work to ensure that AI-generated contributions to the research endeavor do not supersede or otherwise hamper the credibility of the contributions from community members and their representatives. While AI tools are often used to obtain meaning from large datasets, in the participatory public health research field, it is typically the residents themselves who can best provide meaning and context to the data being collected and interpreted.

A mitigation example is that the addition of community advisory experts, panels, and similar oversight bodies can help ensure that the use of AI enriches but does not overshadow either the scientific process itself or scientists and the public’s participation in that process. It will be particularly valuable to include the expertise of ethicists familiar with AI who can identify potential areas of concern and help build appropriate safeguards.

### Ensuring That Scientific Competencies are Enhanced Rather Than Diminished as a Consequence of the AI “Revolution”

With the rapid expansion and ease of use of ChatGPT and other LLM tools across a range of scientific and educational fields, there have been growing concerns related to the detrimental effects that the overuse of such tools can have on current and future generations of scientists and the public. These concerns include the real danger that regular substitution of LLM-generated written and oral communication in science, as well as across other facets of life, could significantly diminish cognitive competencies and the creative and innovative thinking that is the foundation of the scientific process itself. It is clear, at this early stage of generative AI use in participatory science as well as in other scientific areas, that carefully created guidelines for AI usage in all aspects of scientific education and training should be developed and put into place to ensure LLMs and other AI tools support, rather than replace, the facilitation and participation of community-engaged research endeavors.

A mitigation example is described here. A year-long deliberative process by Stanford University’s Department of Epidemiology & Population Health has resulted in a clear set of early principles and guidelines for the use of generative AI tools in one of its major education programs. The guidelines have helped to diminish ambiguity among students and faculty while reinforcing the importance of human-centered educational activities in growing the cognitive competencies of vital importance to the advancement of science. In addition to clear guidelines for AI use and requirements that scientists at all levels of training and experience review their data and methods for accuracy, other suggestions include the further development of field-specific scientific standards for scientific literacy and competence independent of AI usage.

It is also important to ensure that scientific data collection and analysis activities themselves are not in some way diminished or curtailed through overdependence on AI tools. As noted earlier, the judicious use of certain AI tools in the participatory science area may help to enrich and expand the insights and impacts gained through participation by residents, decision makers, and scientists working together. However, it is critical that such AI tools undergo thorough empirical testing to determine when, how, and with whom they can enhance rather than impede scientific knowledge and insights. As one example, the “Our Voice” research team is conducting initial experiments to better determine how the use of “text-to-image” generative AI may affect the ability of citizen scientists to better visualize and understand the proposed health-promoting environmental changes drawn from their data. Among the populations for which such tools are being systematically tested are older US women and school children living in the El Pozón neighborhood of Cartagena, Colombia, which was highlighted earlier. In a second example, a recent “Our Voice” citizen science project conducted in Bogotá, Colombia has illustrated how resident-collected data that were augmented via virtual reality tools can ignite compelling discussions and solution building among participants, scientists, and local stakeholders [22].

The multidimensional mixed-methods data collection methods, sources, and analyses required by the types of participatory scientific methods described in this Viewpoint also may serve as a means for preventing the diminution of cognitive competencies. For example, we have found that the increased complexity of the multilevel, mixed-methods data acquired when microscale (eg, citizen scientists’ “lived experiences”) and macroscale data sources are integrated requires an increased interdisciplinary understanding of both these different forms of data and more intricate and multifaceted data analysis methods, which can serve to enhance cognitive competencies [21].

## Discussion and Conclusions

Citizen science has been shown to be effective in positively engaging diverse groups of community members in meaningful, action-oriented research [17]. Such participatory methods can help to lessen both mistrust in science and civic disengagement, which are trends that threaten to impair advances in public health. Can the judicious applications of various AI tools serve to amplify the potential of participatory science for reducing these trends while broadening meaningful scientific advances in public health? Given that it is clear that the AI “genie” is out of the bottle and will likely have substantial impacts across virtually all aspects of science, it is critical that we meet the AI challenge head on. The inevitable integration of AI into participatory research methods must be thoughtfully managed in order to amplify, and not replace, the collective documentation of human lived experiences, and to ensure that the applications of such data to affect change truly reflect the priorities of local communities. We believe that the time is ripe to systematically evaluate the potential of AI beyond current individual-level applications by exploring its utility as a community aid for advancing public health. At the same time, it is imperative that we continually keep in mind the potential risks accompanying its use, especially among underserved, under-resourced communities. We call on the research community to consider such challenges proactively and empirically over the coming years, finding innovative ways to mitigate the risks while testing the impacts of AI on trust in science, civic engagement, knowledge advancement, and community-driven change.

## Abbreviations

AI: artificial intelligence
LLM: large language model
PPSR: public participation in science research

## References

[1] (2024). The Worlds I See: Curiosity, Exploration, and Discovery at the Dawn of AI.

[2] Kennedy B, Tyson A (2023). Americans’ Trust in Scientists, Positive Views of Science Continue to Decline.

[3] Achterberg P, de Koster W, van der Waal J (2017). A science confidence gap: Education, trust in scientific methods, and trust in scientific institutions in the United States, 2014. Public Underst Sci.

[4] Epstein Z, Sirlin N, Arechar A, Pennycook G, Rand D (2023). The social media context interferes with truth discernment. Sci Adv.

[5] Nasr N (2021). Overcoming the discourse of science mistrust: how science education can be used to develop competent consumers and communicators of science information. Cult Stud Sci Educ.

[6] Alsan M, Wanamaker M (2018). Tuskegee and the health of black men. Q J Econ.

[7] Garrison NA (2013). Genomic justice for native Americans: impact of the Havasupai case on genetic research. Sci Technol Human Values.

[8] Bazargan M, Cobb S, Assari S (2021). Discrimination and medical mistrust in a racially and ethnically diverse sample of California adults. Ann Fam Med.

[9] Kannan VD, Veazie PJ (2023). US trends in social isolation, social engagement, and companionship ⎯ nationally and by age, sex, race/ethnicity, family income, and work hours, 2003-2020. SSM Popul Health.

[10] Byrne M (2023). The disruptive impacts of next generation generative artificial intelligence. Comput Inform Nurs.

[11] Haywood BK, Besley JC (2014). Education, outreach, and inclusive engagement: Towards integrated indicators of successful program outcomes in participatory science. Public Underst Sci.

[12] Mintz E, Couch J (2022). Biomedical citizen science at the National Institutes of Health. Citiz Sci.

[13] Skarlatidou A, Haklay M, Hoyte S, van Oudheusden M, Bishop IJ (2024). How can bottom-up citizen science restore public trust in environmental governance and sciences? Recommendations from three case studies. Environ Sci Policy.

[14] King AC, Winter SJ, Chrisinger BW, Hua J, Banchoff AW (2019). Maximizing the promise of citizen science to advance health and prevent disease. Prev Med.

[15] Allf BC, Cooper CB, Larson LR (2022). Citizen science as an ecosystem of engagement: implications for learning and broadening participation. Bioscience.

[16] King AC, King DK, Banchoff A (2020). Employing participatory citizen science methods to promote age-friendly environments worldwide. Int J Environ Res Public Health.

[17] King AC, Odunitan-Wayas FA, Chaudhury M (2021). Community-based approaches to reducing health inequities and fostering environmental justice through global youth-engaged citizen science. Int J Environ Res Public Health.

[18] English PB, Richardson MJ, Garzón-Galvis C (2018). From crowdsourcing to extreme citizen science: participatory research for environmental health. Annu Rev Public Health.

[19] King AC (2024). Our Voice Global Citizen Science Research Initiative.

[20] Ceccaroni L, Bibby J, Roger E (2019). Opportunities and risks for citizen science in the age of artificial intelligence. Citiz Sci.

[21] Soltani S, Hinman JA, Blanco-Velazquez I (2023). Bringing micro to the macro: how citizen science data enrich geospatial visualizations to advance health equity. J Maps.

[22] Guevara-Aladino P, Sarmiento OL, Rubio MA (2024). Urban care for unpaid caregivers: community voices in the care block program, in Bogotá, Colombia. J Urban Health.

[23] Hsu YC, Verma H, Mauri A, Nourbakhsh I, Bozzon A (2022). Empowering local communities using artificial intelligence. Patterns (N Y).

[24] Garba-Sani Z, Farincacci-Roberts C, Essien A (2024). AI: A new framework for advancing health equity in health care AI. Health Aff.

[25] AI4ALL webpage. https://ai-4-all.org.

